# Protocol for the Study of Heart and Renal Protection-Extended Review: Additional 5-Year Follow-up of the Australian, New Zealand, and Malaysian SHARP Cohort

**DOI:** 10.1177/2054358119879896

**Published:** 2019-10-14

**Authors:** Louisa Sukkar, Ben Talbot, Min Jun, Erika Dempsey, Robert Walker, Lai Hooi, Alan Cass, Meg Jardine, Martin Gallagher

**Affiliations:** 1 The George Institute for Global Health, Newtown, Australia; 2 Faculty of Medicine and Health, The University of Sydney, NSW, Australia; 3 Dunedin School of Medicine, University of Otago, Dunedin, New Zealand; 4 Hospital Sultanah Aminah, Johor Bahru, Malaysia; 5 Menzies School of Health Research, Casuarina, NT, Australia

**Keywords:** chronic renal insufficiency, disease progression, follow-up studies, income, myocardial infarction, poverty, statins

## Abstract

**Background:**

There are limited studies on the effects of statins on outcomes in the moderate chronic kidney disease (CKD) population and their trajectory to end-stage kidney disease.

**Objective:**

To examine the long-term effects of lipid-lowering therapy on all-cause mortality, cardiovascular morbidity, CKD progression, and socioeconomic well-being in Australian, New Zealand, and Malaysian SHARP (Study of Heart and Renal Protection) trial participants—a randomized controlled trial of a combination of simvastatin and ezetimibe, compared with placebo, for the reduction of cardiovascular events in moderate to severe CKD.

**Design:**

Protocol for an extended prospective observational follow-up.

**Setting:**

Australian, New Zealand, and Malaysian participating centers in patients with advanced CKD.

**Patients:**

All SHARP trial participants alive at the final study visit.

**Measurements:**

Primary outcomes were measured by participant self-report and verified by hospital administrative data. In addition, secondary outcomes were measured using a validated study questionnaire of health-related quality of life, a 56-item economic survey.

**Methods:**

Participants were followed up with alternating face-to-face visits and telephone calls on a 6-monthly basis until 5 years following their final SHARP Study visit. In addition, there were 6-monthly follow-up telephone calls in between these visits. Data linkage to health registries in Australia, New Zealand, and Malaysia was also performed.

**Results:**

The SHARP-Extended Review (SHARP-ER) cohort comprised 1136 SHARP participants with a median of 4.6 years of follow-up. Compared with all SHARP participants who originally participated in the Australian, New Zealand, and Malaysian regions, the SHARP-ER participants were younger (57.2 [48.3-66.4] vs 60.5 [50.3-70.7] years) with a lower proportion of men (61.5% vs 62.8%). There were a lower proportion of participants with hypertension (83.7% vs 85.0%) and diabetes (20.0% vs 23.5%).

**Limitations:**

As a long-term follow-up study, the surviving cohort of SHARP-ER is a selected group of the original study participants, which may limit the generalizability of the findings.

**Conclusion:**

The SHARP-ER study will contribute important evidence on the long-term outcomes of cholesterol-lowering therapy in patients with advanced CKD with a total of 10 years of follow-up. Novel analyses of the socioeconomic impact of CKD over time will guide resource allocation.

**Trial Registration:**

The SHARP trial was registered at ClinicalTrials.gov NCT00125593 and ISRCTN 54137607.

## Abrégé

**Contexte:**

On trouve peu d’études faisant état de l’effet des statines sur les issues des patients atteints d’insuffisance rénale chronique (IRC) modérée et sur leur évolution vers l’insuffisance rénale terminale (IRT).

**Objectif:**

Observer les effets à long terme d’un traitement hypolipidémiant sur la mortalité toutes causes, la morbidité cardiovasculaire, la progression de l’IRC et le mieux-être socioéconomique des participants australiens, néo-zélandais et malaisiens, à l’essai SHARP; un essai contrôlé à répartition aléatoire qui portait sur l’effet comparatif d’une combinaison de simvastatine et d’ézétimibe, ou d’un placebo, sur la réduction des événements cardiovasculaires en contexte d’IRC modérée à grave.

**Plan de l’étude:**

Il s’agit d’un protocole pour un suivi prospectif et observationnel prolongé.

**Cadre:**

Les centres d’Australie, de Nouvelle-Zélande et de Malaisie traitant des patients atteints d’IRC de stade avancé et participant à l’essai SHARP.

**Sujets:**

Tous les participants à l’essai SHARP encore vivants lors de la dernière visite de l’étude.

**Mesures:**

Les principaux résultats ont été mesurés par autodéclaration des participants et vérifiés auprès des données administratives de l’hôpital. Les résultats secondaires ont été mesurés à l’aide d’un questionnaire validé évaluant la qualité de vie liée à l’état de santé, une enquête économique de 56 questions.

**Méthodologie:**

Les participants ont été suivis tous les six mois en alternant les visites en clinique et les entretiens téléphoniques, jusqu’à cinq ans après la dernière visite prévue lors de l’essai SHARP. On a procédé au couplage des données avec les registres de santé d’Australie, de Nouvelle-Zélande et de Malaisie.

**Résultats:**

La cohorte SHARP-ER était constituée de 1 136 participants à l’essai SHARP et la durée de suivi médiane était de 4,6 ans. En comparaison de l’ensemble des patients ayant participé à l’essai SHARP en Australie, en Nouvelle-Zélande et en Malaisie, la cohorte SHARP-ER était plus jeune (57,2 [48,3-66,4] contre 60,5 [50,3-70,7] ans), comptait moins d’hommes (61,5 % contre 62,8 %) et présentait une plus faible proportion de patients hypertendus (83,7 % contre 85,0 %) ou diabétiques (20,0 % contre 23,5 %).

**Limites:**

Puisqu’il s’agit d’une étude de suivi à plus long terme, la cohorte de survivants (SHARP-ER) constitue un groupe choisi à partir de l’ensemble des participants à l’essai initial, ce qui pourrait limiter la généralisabilité des résultats.

**Conclusion:**

L’étude SHARP-ER, avec un suivi total sur dix ans, apportera des informations importantes sur les effets à long terme d’un traitement hypolipidémiant chez les patients atteints d’IRC de stade avancé. De nouvelles analyses des impacts socioéconomiques de l’IRC au fil du temps éclaireront l’affectation des ressources.

## What was known before

In people with moderate to severe kidney disease, over a median follow-up of 4.9 years, a combination of simvastatin and ezetimibe, compared with placebo, produced a 17% proportional reduction in major atherosclerotic events (nonfatal myocardial infarction or coronary death, nonhemorrhagic stroke, or any arterial revascularization procedure).

## What this adds

This study aims to extend the follow-up of these individuals to assess the long-term outcomes of statin therapy as kidney disease declines. This study will also provide novel understandings of the economic burden of chronic kidney disease for patients and their families.

## Introduction

Chronic kidney disease (CKD) is a key element of the increasing global burden of chronic diseases.^1^ The increasing prevalence of CKD has been well documented,^2,3^ as has its association with both cardiovascular disease and premature death.^4^ Cardiovascular disease is the leading cause of death in people with CKD, and its prevalence rises with declining kidney function. Importantly, individuals with mild to moderate CKD are more likely to die from cardiovascular causes than develop end-stage kidney disease, the final stage of CKD.^5^ Despite this, there are limited data from large-scale randomized trials on treatments that can slow or halt kidney disease progression or prevent cardiovascular events.

Several randomized placebo-controlled trials have tested the effects of lowering low-density lipoprotein cholesterol (LDL cholesterol) with statin-based therapy in patients with CKD.^6-9^ The Study of Heart and Renal Protection (SHARP) is the largest such study, having randomized 9270 participants with moderate to severe kidney disease in 18 countries. In SHARP, compared with placebo, combination therapy with simvastatin 20 mg and ezetimibe 10 mg yielded an average LDL cholesterol reduction of 0.85 mmol/L (SE = 0.02) over a median follow-up of 4.9 years, producing a 17% proportional reduction in the key prespecified outcome of major atherosclerotic events (MAE) (nonfatal myocardial infarction or coronary death, nonhemorrhagic stroke, or any arterial revascularization procedure) (rate ratio [RR] = 0.83; 95% confidence interval [CI] = 0.74-0.94; *P* = .0021).^9^

Long-term follow-up of efficacy and safety in randomized trials of statins in other populations has demonstrated continuing benefits.^10-14^ However, there are currently only limited examples of such extended follow-up in patients with CKD.^13,15,16^ Extended follow-up of the SHARP cohort offers a unique and valuable resource to further characterize the impact of LDL cholesterol lowering on cardiovascular events, as well as explore the factors associated with CKD progression, and the long-term safety of lipid lowering in those with CKD. To this end, the SHARP Post-Trial Follow-Up (PTFU) study is being undertaken in many of the original countries that participated in SHARP and will determine the long-term effects of 4.9 years of median exposure to simvastatin plus ezetimibe or matching placebo among surviving SHARP participants in relation to major atherosclerotic and major vascular events (MVE); progression to end-stage renal disease (defined as the need for long-term dialysis or renal transplantation) among patients not on maintenance dialysis at randomization to simvastatin plus ezetimibe versus placebo in SHARP; and long-term safety, through assessment of site-specific incident cancers (other than nonmelanoma skin cancer) and mortality by cause.

The SHARP-Extended Review (SHARP-ER) study is part of this broader international initiative and will additionally explore the social and economic impact of CKD on individuals and their household. The SHARP-ER study methods will form the main focus of this article.

## Methods

### Design

The SHARP-ER study is a longitudinal cohort study, extending the follow-up of participants in participating centers in Australia, New Zealand, and Malaysia who were alive at the end of the SHARP trial.

### SHARP Trial

**Figure A1.**
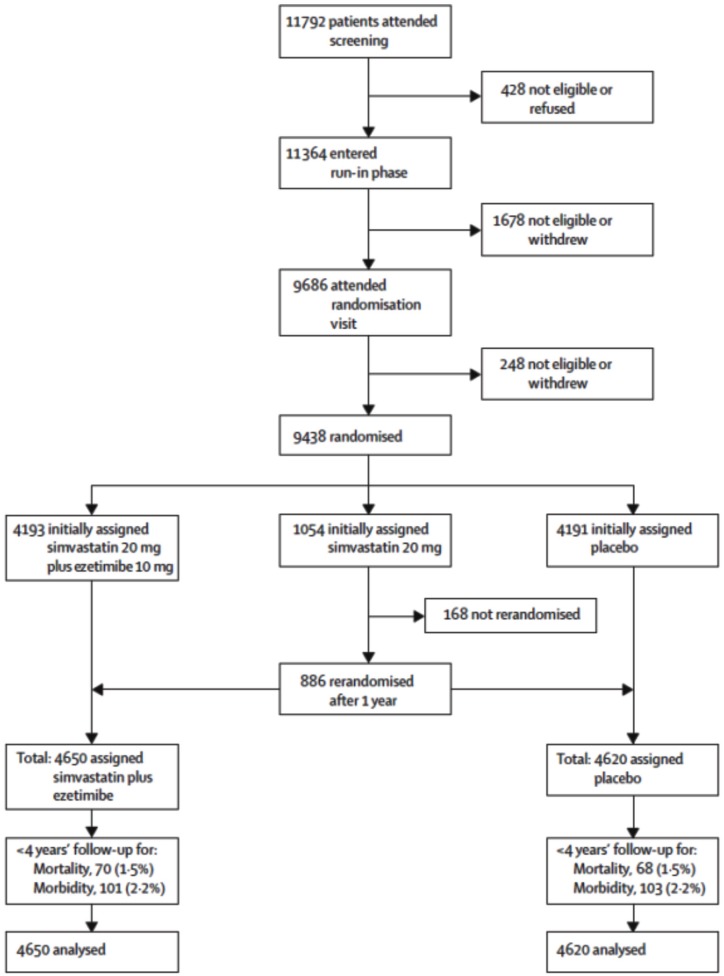
SHARP trial profile.

Details of the recruitment of participants and the study design have been published previously.^9,17^ In brief, 9270 participants aged 40 years or older with CKD (defined as at least 1 measurement of serum creatinine of at least 150 μmol/L in men or 130 μmol/L in women) with no known history of myocardial infarction and coronary revascularization were enrolled between 2003 and 2006 in 18 countries. Participants were randomized in the ratio of 4:4:1 to a combination of simvastatin and ezetimibe, matching placebo, or simvastatin 20 mg alone (Figure A1). Those allocated to simvastatin alone were re-randomized after 1 year to one of the other 2 comparison arms. After initial randomization, participants were followed up in study clinics at 2 and 6 months, and then every 6 months for at least 4 years. At each of these visits, information was recorded on all serious adverse events. A double-dummy method ensured that participants and staff remained unaware of treatment allocation. Although SHARP participants were given the option to discover their treatment after the completion of the original SHARP study, fewer than 3% of participants exercised this option in the global SHARP cohort.

### SHARP Post-Trial Follow-Up

The SHARP-PTFU seeks to provide long-term follow-up of the global cohort of SHARP participants alive at the end of the SHARP trial. It will assess the primary and secondary outcomes of SHARP over an additional 5 years with participating centers using a number of methods, including post-trial questionnaires and linkage to routinely collected national data sets (eg, hospital admission data, cancer and mortality data).

As a component of SHARP-PTFU, the SHARP-ER study will contribute primary and secondary post-trial outcome data to the PTFU, with the differences in the 2 initiatives summarized in Table 1.

**Table 1.** Differences Between SHARP-ER Study and the Global SHARP-PTFU Study.

	SHARP-ER study	SHARP-PTFU study
Population	SHARP survivors in Australia, New Zealand, and Malaysia	All SHARP survivors
Outcome measures	Face-to-face visits Telephone interviews Study questionnaires Linkage to registry data	Linkage to registry data
Outcomes	Major atherosclerotic events^a^ Major vascular events^b^ Renal outcomes^c^ Socioeconomic outcomes^d^	Major atherosclerotic events Major vascular events Renal outcomes
Follow-up	5 years	5 years with ongoing linkages planned

*Note.* SHARP = Study of Heart and Renal Protection; SHARP-ER = Study of Heart and Renal Protection-Extended Review; PTFU = Post-Trial Follow-Up

a Major atherosclerotic events defined as coronary death, myocardial infarction, nonhemorrhagic stroke, or any revascularization procedure (excluding vascular access surgery for dialysis).

b Major vascular events defined as hemorrhagic stroke and noncoronary death.

c Renal outcomes defined as initiation of long-term renal replacement therapy or renal transplantation.

d Socioeconomic outcomes will include an assessment of (1) illness-related catastrophic expenditure, (2) illness-related poverty, and (3) economic hardship.

The SHARP-ER commenced recruitment in August 2012. All participants alive at the final SHARP study visit in participating centers in Australia, New Zealand, and Malaysia (August 2010) who were not previously documented as having withdrawn consent were eligible for inclusion in SHARP-ER. Exclusion criteria for SHARP-ER were the presence of concomitant major illness that would limit the participant’s follow-up (in the opinion of the treating physician), a high likelihood that the participant would not adhere to follow-up, and inability to provide informed consent for reasons of mental or physical incapacity.

The study was conducted in accordance with the approved study protocol, the principles of the “Declaration of Helsinki,” and the laws and regulations of the relevant countries. All participating centers obtained independent ethics approval prior to study commencement.

### Study Procedures

The SHARP-ER study did not involve allocation to further study treatment. The nature of any cholesterol treatment used by participants following the end of the SHARP Study was measured by questionnaire and data linkage.

The vital status of all SHARP study participants at 18 to 24 months after their final study visit was determined through medical records, direct contact with medical staff (renal physicians and general practitioners), and death registries.

Consenting participants were followed up with 3 face-to-face visits at 18 to 24 months, 3.5 years, and 5 years, followed by the final SHARP Study visit. In addition, there were 6-monthly follow-up telephone calls in between these visits (Table 2).

**Table 2.** Study of Heart and Renal Protection-Extended Review Study Schedule.

Time since completion of final SHARP study visit	Participant enrollment (screening)	Visit 1 18-24 mo	Telephone call 30 mo	Telephone call 36 mo	Visit 2 42 mo	Telephone call 48 mo	Telephone call 54 mo	Visit 3 60 mo
Prior written consent	x	x						
Survival status	x	x	x	x	x	x	x	x
Physical signs		x			x			x
SHARP primary events (major atherosclerotic events)		x	x	x	x	x	x	x
Subsidiary study outcomes		x	x	x	x	x	x	x
Biochemistry		x			x			x
Hematology		x			x			x
Socioeconomic questionnaire		x						x
Registry linkage								x

The data collection included the following:

- Physical signs: weight, height, and blood pressure;
- Medication usage: including the use of lipid-lowering and antiplatelet medications;
- Assessment of primary and secondary SHARP-ER study outcomes: including admissions to hospital, and requirements for chronic dialysis or kidney transplant. These outcomes were ascertained by participant self-report at telephone interview or at the individual patient visit. This was further verified using discharge summaries from the treating hospitals;
- Biochemistry: serum and urine specimens obtained as part of routine care within 3 months either side of the date of study visit to characterize progression of kidney disease;
- Hematology: blood specimens obtained as part of routine care within 3 months either side of the date of face-to-face study visit;
- Questionnaire: quality of life, health services usage, and socioeconomic impact of CKD administered using study questionnaires at visits 1 and 3.

The study questionnaire was developed using questions drawn from the existing validated tools to evaluate health-related quality of life (HR-QoL)^18^ and the social,^19^ cognitive, and emotional impacts of kidney disease.^20^ The HR-QoL was measured through telephone interview by a central interviewer (blinded to SHARP study allocation) at the initial visit, followed by assessments at 3.5 and 5 years of follow-up. These interviews used the EuroQOL 5 dimensions questionnaire (EQ-5D) and a Health Services Usage Questionnaire. In addition, a 56-item detailed economic survey was used to assess the socioeconomic impact of CKD. This included an assessment of (1) out-of-pocket expenditure on illness not covered by insurance, such as expenditure on health care, medications, investigations, and paid care; (2) economic hardship, defined as an inability to make necessary household payments, such as housing, energy, food, and health care costs, or requiring assistance to meet such costs^21^; (3) household income in the past 12 months, measured against the median income levels obtained from National Statistical Bureau.

Where available, data linkage using registries was used as secondary ascertainment for mortality (using national death registries and Australian Institute of Health and Welfare’s National Death Index^22^) and dialysis commencement (using renal replacement therapy [RRT] registries: the Australian and New Zealand Dialysis and Transplant Registry [ANZDATA; which captures 99% of all participants commencing RRT in Australia and New Zealand]^23^ and the Malaysian National Renal Registry). In addition, consenting participants were linked to the Medicare Benefits Schedule (MBS)^24^ and the Pharmaceutical Benefits Scheme (PBS)^25^ in Australia to evaluate the health care costs of CKD and long-term LDL cholesterol–lowering treatment.

A limited assessment of the deceased participants who were alive at SHARP study closure, but died prior to the SHARP-ER study, was also undertaken. This included date and cause of death (from death certificates), and requirement for dialysis in the period between the last assessment for the SHARP study and death.

### Study Outcomes

The primary objective of the SHARP-ER study is to contribute to the description of the long-term effects of SHARP study treatments, as part of the larger PTFU, on MAE (coronary death, myocardial infarction, nonhemorrhagic stroke, or any revascularization procedure [excluding vascular access surgery for dialysis]) and MVE (hemorrhagic stroke and noncoronary death). An important secondary objective of the study is the long-term effects of the SHARP study treatment on rates of CKD progression, defined by initiation of long-term RRT or renal transplantation. Other secondary outcomes included cancer development (excluding nonmelanoma skin cancer) and all-cause mortality. These outcomes will be analyzed using an intention-to-treat analysis

The SHARP-ER study also measured the economic impact of CKD on participants and households at visits 1 and 3. This included a detailed appraisal of (1) the incidence of illness-related catastrophic expenditure, assessed as out-of-pocket illness expenditure exceeding 30% of annual household income over a previous 12-month period^26^; (2) the incidence of illness-related poverty, assessed by a change in reported household income that sees a household transition from above the prevailing national poverty line (country specific) at baseline to below, over a previous 12-month period; and (3) the incidence of economic hardship, defined as perceived economic difficulties that arise as a result of chronic illness, which alters the way people affected by illness live and manage their conditions.^27^ The economic impact of disease will be measured as a difference between visit 1 and visit 3. Economic impact will also be compared across different CKD stages, which will enable an appreciation of the changing costs and economic impact associated with disease progression.

### Statistical Analysis

Continuous variables will be reported as means with standard deviations for variables with approximately symmetric distributions and as median and interquartile ranges (IQRs) for those with skewed distributions. Study outcomes, including economic outcomes, will be assessed according to CKD category tested by linear regression analysis and logistic, Cox, or Poisson regression analysis (to estimate odds ratios, hazard ratios, and rates, respectively, with their corresponding 95% CIs), as appropriate. Multivariable models will be constructed adjusting for baseline variables, including country of participant, sociodemographic information (age, sex, body mass index, ethnicity, income, and insurance status), laboratory measurement results (estimated glomerular filtration rate, urinary albumin measurements, hematology), and comorbid conditions (diabetes mellitus, hypertension, atrial fibrillation, cardiac failure). Interaction terms between CKD category and relevant variables will be included to test for effect modification by CKD. In all time-to-event analyses, participants will be followed from baseline until the date of the outcome, death, or study completion. Analysis of the economic outcomes will use multivariate logistic regression models analogous to previous work in this area^28^ Statistical analyses will be performed with SAS 7.11 (SAS Institute, Cary, NC, USA) and Stata software (release 13; StataCorp, College Station, TX, USA). A 2-sided *P* < .05 will be considered statistically significant.

## Results

**Figure 1.**
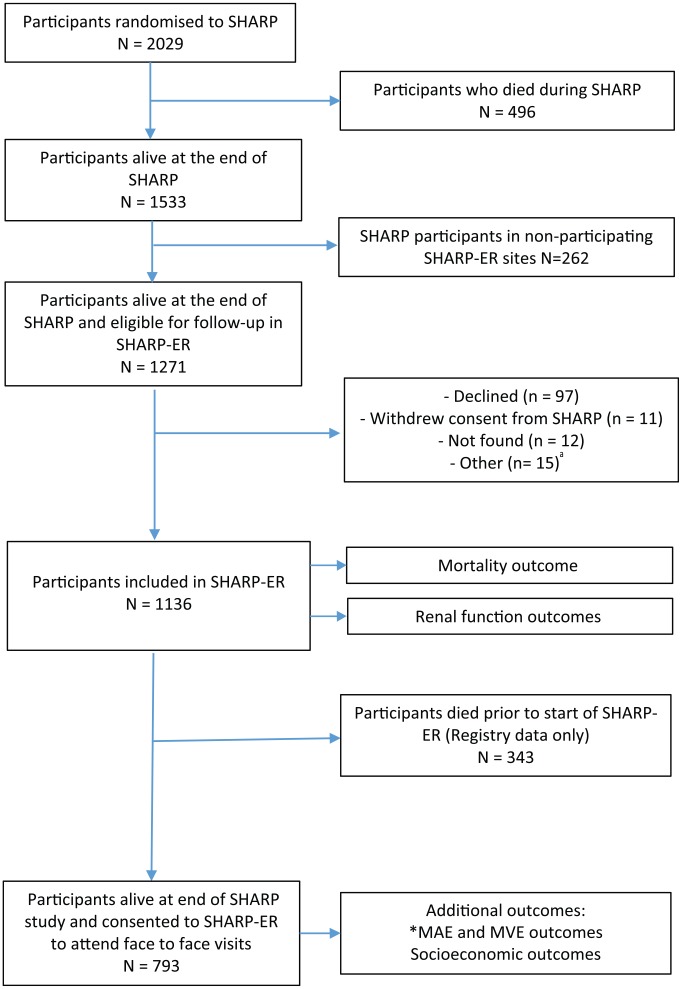
Flow diagram of participants available for SHARP-ER outcomes. *Note.* SHARP = Study of Heart and Renal Protection; SHARP-ER = Study of Heart and Renal Protection-Extended Review. ^a^Other: noncompliance to study visits n = 8; physician discretion n = 7. *MAE = major atherosclerotic events; MVE = major vascular events.

Of the original 58 SHARP study sites in Australia, New Zealand, and Malaysia, 44 sites agreed to participate in SHARP-ER. Within these sites there were a total of 1271 participants eligible for inclusion, of whom 1136 (89.4%) were included in the final SHARP-ER cohort. A proportion who died were entered according to the SHARP study consent (Figure 1). Compared with the original SHARP participants in Australia, New Zealand, and Malaysia at the beginning of SHARP, SHARP-ER participants were younger (median age = 57.2 [IQR = 48.3-66.4] vs 60.5 [50.3-70.7]) and had a lower proportion with comorbid diabetes (20.0% vs 23.5%). All other baseline characteristics including blood pressure, renal function, and lipid profile were similar (Table 3). The proportion of participants on RRT at the beginning of SHARP was also similar between the 2 cohorts.

**Table 3.** Baseline Characteristics of the Australian, New Zealand, and Malaysian (AUS/NZ/MYL) SHARP and SHARP-ER Participants at SHARP Commencement.

Characteristic	AUS/NZ/MYL SHARP participants (N = 2029)	SHARP-ER participants (N = 1136)
Sex, No. (%)		
Men	1274 (62.8)	699 (61.5)
Women	755 (37.2)	437 (38.5)
Age (years)		
Median (IQR)	60.5 (50.3-70.7)	57.2 (48.3-66.4)
No. (%)		
40-49	495 (24.4)	338 (29.8)
50-59	498 (24.5)	333 (29.3)
60-69	496 (24.5)	263 (23.2)
70+	540 (26.6)	202 (17.8)
Comorbidities, No. (%)^a^		
Diabetes	477 (23.5)	227 (20.0)
Hypertension	1725 (85.0)	951 (83.7)
Blood pressure,^b^ mean (SD)		
Systolic blood pressure, mm Hg	141 (23.0)	140 (23.0)
Diastolic blood pressure, mm Hg	79 (13.0)	80 (12.0)
Renal status, No. (%)		
Not on renal replacement therapy (CKD)	1308 (64.5)	751 (66.1)
On renal replacement therapy	721 (35.5)	385 (33.9)
Laboratory values		
eGFR, mL/min/1.73 m^2c^		
45-59	28 (2.2)	21 (2.8)
30-44	345 (26.5)	211 (28.1)
15-29	647 (49.7)	372 (49.6)
<15	283 (21.7)	146 (19.5)
Albumin-to-creatinine ratio measurements, No. (%)^d^		
<30 mg/g	254 (19.0)	140 (18.6)
30-300 mg/g	495 (37.1)	290 (38.5)
>300 mg/g	586 (43.9)	324 (43.0)
Mean lipid, mmol/L, mean (SD)^e^		
Total cholesterol	4.9 (1.1)	4.9 (1.1)
LDL	2.8 (0.8)	2.8 (0.8)
HDL	1.0 (0.3)	1.1 (0.3)
Triglycerides	2.5 (1.8)	2.6 (1.7)
Country		
Australia	1043 (51.4)	468 (41.2)
New Zealand	285 (14.1)	133 (11.7)
Malaysia	701 (34.6)	535 (47.1)

*Note.* SHARP = Study of Heart and Renal Protection; IQR = interquartile range; CKD = chronic kidney disease; LDL = low-density lipoprotein; HDL = high-density lipoprotein; eGFR = estimated glomerular filtration rate.

a Available for all participants (n = 1136/2029).

b Systolic blood pressure was available for n = 1134/2026 participants; diastolic blood pressure was available for n = 1133/2025.

c eGFR calculated using the modified renal diet (MDRD) equation. eGFR was calculated for all participants not on renal replacement therapy with available data (n= 750/1303).

d The albumin-to-creatinine ratio was measured in milligrams of albumin and grams of creatinine; it was available for n = 754/1335.

e Lipid values were available for n = 757/1941.

## Discussion

The SHARP trial was a large-scale randomized controlled trial, which assessed the effects of LDL lowering in patients with moderate to severe CKD. In SHARP, allocation to combination therapy with simvastatin plus ezetimibe over a median of 4.9 years reduced the incidence of MAE without an increase in any of the prespecified safety outcomes. Long-term follow-up of efficacy and safety in randomized trials of statin-based LDL-lowering therapy in other populations has demonstrated continuing benefits on vascular events and reassuring safety for nonvascular events such as cancer.^11-14^ While extended follow-up of patients in the 4D (Die Deutsche Diabetes Dialyse) and Assessment of LEscol in Renal Transplantation (ALERT) trials of lipid lowering in those with CKD has been done previously, these studies only included patients on dialysis or who had undergone a renal transplant, meaning that there is a paucity of evidence for the long-term effects of LDL lowering in those with moderate to severe predialysis kidney disease.^15,16^

To address this issue, the SHARP-PTFU study will assess the long-term effects of lowering LDL cholesterol on first MAE, progression of renal disease, and long-term safety outcomes among surviving SHARP participants. The SHARP-ER study conducted in Australia, New Zealand, and Malaysia is part of this broader international initiative and followed surviving SHARP participants for a further 5 years with face-to-face visits and telephone contact at 6-monthly intervals with supplementary data linkage to administrative and health registries and benefit schemes. In addition, it has collected information to assess the social and economic impact of CKD on individuals and their household.

Extended follow-up of such a large clinical trial is important because the SHARP trial might have been too short to detect any latent carcinogenic potential of LDL lowering with simvastatin plus ezetimibe. It is also valuable in providing data on the determinants of renal disease progression, as CKD often has a gradual and slowly progressive disease course.

The linkage of the SHARP-ER follow-up to registries and administrative data sets will also enable a more detailed understanding of chronic disease, as well as facilitating hypothesis generation for future research and providing valuable data on medication use along with the uptake of guideline-recommended therapy in a population where mitigation of cardiovascular risk is of paramount importance. The information gained will help to identify the treatment gaps and ascertain the factors which predispose to their reduced uptake, aiding in more efficient health resource allocation.

The SHARP-ER study will provide detailed measurements of the economic impacts of CKD from a patient perspective. Most studies that estimate out-of-pocket costs only quantify direct costs for treatment and medications, overlooking the considerable financial burden associated with self-management, including medically related transport, home-care assistance, illness-related modifications (eg, for home dialysis setup), and assistive devices. Moreover, limited data are available, which quantify personal and household economic impact more broadly with measures such as economic hardship and financial distress. The SHARP-ER attempts to overcome these deficiencies using a patient questionnaire, at 2 time points (visits 1 and 3), which include questions pertaining to household income, financial hardships (difficulty paying utility bills, mortgage repayments), as well as direct health care costs to the individual. With data on the stage of CKD, it will permit a deeper understanding of how the financial pressures vary over the duration of this chronic disease, helping to guide future resource allocation to areas of greatest patient need.

Limitations of this cohort study include the ability to generalize the findings to the wider CKD population given that participants needed to survive to enter the post-trial long-term follow-up. Despite this, the baseline characteristics of those who survived and were eligible to enter SHARP-ER were similar to those of the original SHARP cohort in the region, suggesting the SHARP-ER cohort to be representative of the wider SHARP cohort. To minimize the burden of additional travel and potential cost, laboratory results performed as part of routine care were used in the data collection. This has limitations due to variability between laboratories regarding measuring methods and normal ranges within a country and between different countries.

## Conclusions

In conclusion, the SHARP-ER study is the collection of detailed data for a well-characterized cohort with moderate to severe CKD. It will allow for reporting of outcomes of MAE and MVE, rates of CKD progression, rates of cancer development (excluding nonmelanoma skin cancer), and all-cause mortality, medication usage, and socioeconomic impacts. Data for many of these outcomes will be available for a 10-year period (5 years of SHARP trial data and a further 5 years of follow-up with SHARP-ER), enabling analysis of recurrence of events and an unprecedented understanding of the burden of morbidity over time.

## Appendix

### SHARP-ER Study

#### Regional Coordination

*The George Institute for Global Health, Sydney*: A. Cass, M. Gallagher, Graham Hillis, J. Lee, E. Dempsey, A. Yianni, M. Gorzeman, S. Spratley, E. Ivanova, N. Nath Kumar, W. Ooi, B. Essue, S. Coggan, S. Decollogne, E. Fjalling

### Local Clinical Center

#### Australia

*Albury Base Hospital*: R. Auwardt, P. Cogdell

*Austin Health, Melbourne*: P. Mount, M. Roberts, M. Veenendaal, P. Bisscheroux

*Bundaberg Base Hospital*: P. Miach, D. Booth, C. Arnold

*Cairns Base Hospital*: M. Mantha, S. Green

*Central Northern Adelaide Renal and Transplantation Service (CNARTS)*: T. Elias, S. McDonald, M. Hockley, K. Fisher

*Concord Repatriation Hospital*: S. Sen, S. Hand

*Core Research Group Pty Ltd, Milton*: D. Colquhoun, A. Ferreira-Jardim, H. Morison, L. Williams

*Fremantle Hospital*: P. Ferrari, S. Swaminathan, U. Steinwandel, K. Hollmann, B. Siva

*Gold Coast Hospital*: E. Meagher, T. Titus, H. McEvoy, T. Schmidt, T. Chad

*John Hunter Hospital, Newcastle*: S. Carney, L. Garvey, A. Gillies, T. Brown, Y. Choi

*Launceston General Hospital*: M. Mathew, D. Cooke, S. Smith

*Liverpool Hospital*: M. Suranyi, G. Rayment, J. Wong, M. Wong

*Nambour General Hospital*: N. Gray, A. Pollock, S. Wadham

*Nepean Hospital, Penrith*: R. Wyndham, K. Sud, N. Ubera, P. Murie

*Princess Alexandra Hospital, Woolloongabba*: D. Johnson, C. Hawley, J. Sudak

*Renal Research, Gosford*: S. Roger, L. Bohringer

*Royal Hobart Hospital*: M. Jose, L. Jeffs, G. Kirkland, R. Papatriantafillou, S. Hennessy

*Royal Melbourne Hospital*: E. Pedagogos, M. Farrell, C. Karschimkus, M. Raspudic, N. Toussaint

*Royal North Shore Hospital, St Leonards*: B. Cooper, J. Pearse, A. Mather, H. Tsang, M.G. Wong, C. Weischelberger

*Royal Prince Alfred Hospital, Sydney*: P. Snelling, V. Bielski, S. Sherwood, A. Bisson, M. Barden

*Sir Charles Gairdner Hospital, Perth*: B. Hutchison, H. Herson, S. Pellicano, G. Dogra, W. Lim, D. Chan, H. Moody, N. Boudville

*St Vincent’s Hospital, Fitzroy*: R. Langham, K. Mullins

*Sydney Adventist Hospital*: P. Collett, A. Heath, J. Esplin, K. Sutherland, D. Talafua

*The Canberra Hospital*: G. Talaulikar, P. Johnson

*Westmead Hospital*: G. Rangan, P. Murie, H. Heathwood

*Wollongong/Shellharbour Hospitals*: M. Lonergan, M. Magill, C. Wen

#### Malaysia

*Hospital Ipoh*: C.L. Loh, Norlia K, Y.Y. Lee

*Hospital Kuala Lumpur*: Ghazali A., N. Baskaran, S. Bavanandan, R. Visvanathan, S.L. Wong, Rosnawati Y.

*Hospital Kuala Terengganu*: Zawawi N., Zaiha H, Hindun A.

*Hospital Melaka*: Korina R., Yunaidah A.

*Hospital Pulau Pinang*: L.M. Ong, Rozina G., S.A. Goh, Y.F. Liew, G.L. Teoh

*Hospital Raja Perempuan Zainab II, Kota Bharu*: Wan Hasnul W. H, Norhayati A., Norhayati I., Sukeri M., Zuad F.R.

*Hospital Selayang*: H.S. Wong, C.Y. Goh, B.C. Bee, C. Ramasamy, Rafidah A.

*Hospital Sultanah Aminah, Johor Bahru*: L.S. Hooi, W.J. Liu, Razali O., Haslinah S.

*Hospital Taiping*: I. Vaithilingam, Jaaini A., Faridah L., C.H. Lim

*Hospital Tengku Ampuan Afzan*: Ramli S., Rosnah A.A., C.C. Tam, Ahmad Fuad A.T., Fariz Safhan M.N.

*Hospital Tengku Ampuan Rahimah, Selangor*: C.C. Tan, Shahnaz F.K., Wazir H., Azura H.B.

*Hospital Tuanku Jafa’ar, Seremban*: Lily M., Wan Shaariah M.Y., Faezah I., W.M. Lim, S. Sivathasan, Fuziah Z

*Hospital Umum Sarawak*: C.H.H. Tan, Javelin P., L.S. Ngu, L.W.S. Hii

*University Malaya Medical Center, Kuala Lumpur*: S.K. Lim, K.P. Ng, L.P. Tan, T.C. Keng, Asmalina M.

#### New Zealand

*Auckland City Hospital*: J. Collins, M. Upjohn

*Christchurch Hospital*: D. McGregor, J. Usher

*Dunedin Hospital*: R. Walker, G. Ellis

*Middlemore Hospital, Auckland*: D. Voss, M. Upjohn
